# Complete Genome Sequence of *Mycobacterium ulcerans* subsp. *shinshuense*

**DOI:** 10.1128/genomeA.01050-16

**Published:** 2016-09-29

**Authors:** Mitsunori Yoshida, Kazue Nakanaga, Yoshitoshi Ogura, Atsushi Toyoda, Tadasuke Ooka, Yuko Kazumi, Satoshi Mitarai, Norihisa Ishii, Tetsuya Hayashi, Yoshihiko Hoshino

**Affiliations:** a Department of Mycobacteriology, Leprosy Research Center, National Institute of Infectious Diseases, Higashi-Murayama, Tokyo, Japan; b Department of Bacteriology, Faculty of Medicine Sciences, Kyushu University, Fukuoka, Fukuoka, Japan; c Center for Information biology, National Institute of Genetics, Mishima, Shizuoka, Japan; d Department of Microbiology, Graduate School of Medical and Dental Sciences, Kagoshima University, Kagoshima, Kagoshima, Japan; e The Research Institute of Tuberculosis, Japan Anti-Tuberculosis Association, Kiyose, Tokyo, Japan

## Abstract

*Mycobacterium ulcerans* subsp. *shinshuense* produces mycolactone and causes Buruli ulcer. Here, we report the complete sequence of its genome, which comprises a 5.9-Mb chromosome and a 166-kb plasmid (pShT-P). The sequence will represent the essential data for future phylogenetic and comparative genome studies of mycolactone-producing mycobacteria.

## GENOME ANNOUNCEMENT

*Mycobacterium ulcerans* is a causative agent of Buruli ulcer (1). Buruli ulcer is characterized as a chronic, indolent, necrotizing disease of skin and soft tissue, which produces ulcers followed by scar formation (2). Its first reported case in Japan occurred in 1980 (3, 4). The causative agent was isolated and identified as *M. ulcerans* subsp. *shinshuense* because it was closely related to *M. ulcerans*, although certain distinct differences were observed (3–5). The first clinical isolate (ATCC 33728^T^) is a reference strain of *M. ulcerans* subsp. *shinshuense* (6). Here, we report the complete genome sequence of the chromosome and a large plasmid of ATCC 33728^T^.

The strain was grown on Middlebrook 7H10 agar medium. DNA was purified with a high pure PCR template preparation kit (Roche Diagnostics). The genome sequence was determined using the 454 GS FLX Titanium system (Roche Diagnostics). A total of 889,811 single-end and 115,770 paired-end (8-kb insert) reads were assembled with the GS Assembler software version 2.6 into three scaffolds containing 259 gaps. All gaps, except for one large gap in the plasmid, were closed by sequencing of gap-spanning PCR products using an ABI3130xl DNA sequencer (Applied Biosystems). Illumina 150 × 2 paired-end reads (1,249,780 reads) obtained by a MiSeq sequencer (Illumina) and PacBio reads (47,671 subreads over 15.0 kb) obtained by an RS II system were used for sequence- and assemble-error correction and closing of a large gap in the plasmid. Automated annotation was carried out with the Microbial Genome Annotation Pipeline (MiGAP) (http://www.migap.org). Manual curation of the automatic annotation was performed using the *in silico* molecular cloning (In Silico Biology).

The chromosome of ATCC 33728^T^ is 5,899,681 bp in length with a 65.64% G+C content, and the plasmid pShT-P is 166,617 bp in length with a 62.76% G+C content. The average nucleotide identities were 98.36% to *M. ulcerans* (strain Agy99) and 99.10% to *M. liflandii* (strain 128FXT), indicating their close genetic relatedness and supporting its taxonomic position as a subspecies of *M. ulcerans* (7). The chromosome contains 5,015 predicted protein coding sequences (CDSs), which is much more than those of the *M. ulcerans* strain Agy99 chromosome (4,160). Plasmid pShT-P contains 72 CDSs, whereas 81 CDSs were present in the large plasmid of *M. ulcerans* (8). The total number of insertion sequences is 233 (12 on the plasmid), which is less than that of *M. ulcerans* (324), but more than that of *M. marinum* (50 in strain M) (9). Notably, the number of IS*2606* is significantly less than that of *M. ulcerans* (2 versus 91). The draft genome sequence of ATCC 33728 was previously determined (10), but it contains no plasmid sequence. This is probably due to the deletion of plasmid pShT-P, which frequently occurs during *in vitro* cultivation (10, 11). The genome sequence of *Mycobacterium ulcerans* subsp. *shinshuense* ATCC 33728^T^ will represent the essential data for future phylogenetic and comparative genome studies and will be useful for better understanding the evolution of mycolactone-producing mycobacteria.

### Accession number(s).

This complete genome sequence has been deposited at DDBJ/ENA/GenBank under the accession no. AP017624 to AP017625.
